# The effect of calcium and vitamin D supplementation on obesity in postmenopausal women: secondary analysis for a large-scale, placebo controlled, double-blind, 4-year longitudinal clinical trial

**DOI:** 10.1186/1743-7075-7-62

**Published:** 2010-07-23

**Authors:** Jiapeng Zhou, Lan-Juan Zhao, Patrice Watson, Qin Zhang, Joan M Lappe

**Affiliations:** 1 Key Laboratory of Animal Genetics and Breeding of the Ministry of Agriculture, College of Animal Science and Technology, China Agricultural University, Beijing, China; 2 Osteoporosis Research Center, Creighton University Medical Center, Omaha, NE, USA; 3 Department of Biomedical Sciences, Creighton University Medical Center, Omaha, NE, USA

## Abstract

**Background:**

It is undetermined whether calcium supplementation has an effect on obesity or body composition in postmenopausal women. The purpose of the study is to detect the effect of calcium supplementation on indices of obesity and body composition.

**Methods:**

This is a secondary analysis of data from a population-based, double-blind, placebo-controlled, randomized trial designed to determine the effects of calcium and vitamin D on osteoporotic fractures. The cohort included 1179 postmenopausal women who were randomly assigned into one of three groups: 1) supplemental calcium (1400 mg/d or 1500 mg/d) plus vitamin D placebo (Ca-only group); 2) supplemental calcium (1400 mg/d or 1500 mg/d) plus supplemental vitamin D3 (1100 IU/d) (Ca + D group); or, 3) two placebos (placebo group). After applying the exclusion criteria for this analysis, 870 subjects were included in this study. The primary outcomes for the present study were changes in body mass index, trunk fat, trunk lean, and percentage of trunk fat after calcium supplementation.

**Results:**

Changes in trunk fat, trunk lean, and percentage of trunk fat were significantly different between the calcium intervention groups (Ca-only group or Ca + D group) and the placebo group during the trial (*P *< 0.05). The calcium intervention groups gained less trunk fat and maintained more trunk lean when compared to the placebo group. No significant difference was observed for body mass index between groups.

**Conclusion:**

Calcium supplementation over four years has a beneficial effect on body composition in postmenopausal women.

## Background

An urgent need exists to identify modifiable dietary risk factors for obesity. Obesity has become a major health threat around the world. It is epidemic, and the risk increases with age [1-3]. In fact, it is estimated that ~70% of Americans over 60 years are overweight [4]. Women are more prone to the risk of obesity than men [3]. Elderly women who have excess body fat accumulation face increased risk for coronary heart disease, hypertension, metabolic syndrome, osteoarthritis, diabetes mellitus, and other co-morbidities [5-10].

A body of evidence has emerged to support the hypothesis that dietary calcium plays a role in decreasing the risk of obesity. Cross-sectional studies have shown that calcium intake has a negative correlation with body weight, body mass index (BMI), body fat, and percentage of body fat [11-16]. For example, in the NHANES III survey [17], the odds ratio of being in the highest quartile of body fat was significantly lower if individuals were in the highest quartile of calcium intake. The relative risk of high body adiposity was found to be highest in those with the lowest calcium intake and was progressively lower as calcium intake increased.

Randomized placebo-controlled trials have provided evidence that is supportive of the beneficial effect of high calcium intake on obesity [18-21]. In these trials, greater weight loss and/or fat mass loss were observed in the high-dairy supplemented group compared to the placebo group. Data from the Women's Health Initiative (WHI) Study has provided supportive evidence as well [22]. In this randomized placebo-controlled trial, which involved over 36000 postmenopausal women over an average term of seven years, the calcium plus vitamin D supplemented group experienced smaller weight gain than the placebo group.

While the aforementioned clinical trials indicated a beneficial effect of calcium, two systematic reviews found no evidence of benefit from calcium supplementation on body weight loss [23,24]. Thus, the role of supplemental calcium in preventing obesity remains controversial.

It should be noted that the abovementioned clinical trials were generally conducted in obese [18-20] and/or low-calcium consumers [18-20,22], and that increased dietary calcium was primarily in the form of dairy products [18-21]. In the WHI Study, the phenotype is total body weight [22]. However, weight is a heterogeneous phenotype consisting of fat, lean, and bone mass. Therefore, the change in weight does not always reflect the change in fat.

It is reported that serum 25-hydroxy vitamin D [25(OH)D] is negatively associated with obesity status [25-27]. That is, those subjects who have high serum 25(OH)D levels generally have a lower risk of obesity. However, the relationship between vitamin D intake and obesity is yet undetermined [28,29].

Our population-based study of older women who have a wide range of body size provided an opportunity to assess the effects of calcium, and calcium plus vitamin D supplements on obesity in a large sample over a sustained, four-year period of time. Moreover, this trial included a variety of phenotypes by which to assess the calcium and calcium plus vitamin D effects. Fat mass and lean mass are more homogenous phenotypes in comparison to body weight or BMI. Thus, these phenotypes should be more sensitive to the possible beneficial effects of calcium and calcium plus vitamin D.

## Methods

### Participants

#### 1) The subjects for the 4-year clinical trial

The original cohort included 1179 non-Hispanic white women who were randomly selected from a population of postmenopausal women > 55 years of age in a nine-county rural area of the Midwest. A full-service market research firm randomly selected telephone numbers from all households with listed numbers in the nine-county rural sample area. The firm identified 1180 women meeting the inclusion and exclusion criteria who were willing to participate in a four-year prospective study of calcium and vitamin D supplementation. The participants were enrolled into study between May 2000 and July 2001. The study was approved by the Creighton University Institutional Review Board, and signed informed consent was obtained from each participant. The inclusion criteria for the 1180 subjects were: 1) at least four years since last menses; 2) in generally good health; 3) living independently in the community; and 4) weighing less than 300 pounds. Exclusion criteria included: a history of cancer except for basal and squamous cell skin cancers and other cancers treated curatively over ten years prior to enrollment; history of renal calculi or chronic kidney disease; and, a history of Paget's disease. One woman was excluded after entry, when she disclosed a history of hypoparathyroidism following thyroidectomy and reported having taken 50000 IU of vitamin D daily for the past 25 years.

The subjects were randomly assigned to one of three groups: 1) calcium, consisting of either calcium citrate (1400 mg/d) or calcium carbonate (1500 mg/d) plus a vitamin D placebo (defined as Ca-only group, 445 subjects); Heaney et al. found that, when taken with food, calcium from the carbonate salt is fully as absorbable as from the citrate [30]; 2) calcium plus vitamin D, consisting of calcium (as above) plus 1100 IU cholecalciferol (vitamin D_3_)/d (defined as Ca + D group, 446 subjects); and 3) placebos, consisting of both a vitamin D placebo and a calcium placebo (defined as placebo group, 288 subjects). In the present study, calcium and vitamin D placebo, calcium and vitamin D, or placebos only, were taken three times daily, to be swallowed with meals.

Enrolled subjects were assigned to groups using computer-generated permuted blocks (*n *= 5) randomization scheme. By design (initially for fracture), the two active treatment groups (Ca only and Ca + D group) were each allocated ≈40% (446 of 1180 participants) of the cohort, and the placebo group, 20% (288 of 1180 participants). Of the 1180 women enrolled, 1024 (86.9%) completed the 4-year study. Most withdrawals (*n *= 92) occurred within the first year. Compliance with study medication (both active and placebo) was assessed at 6-month intervals by bottle weight. The average compliance rate (defined as taking ≥ 80% of assigned doses) was 74.4% for the calcium component, and 85.7% for the vitamin D component.

#### 2) Subjects selected for the present study

In order to reduce confounding effects, we established three exclusion criteria for selecting subjects for this analysis. Subjects who had metal prostheses/implants were excluded. Metal prostheses/implants interfere with the accuracy of a DXA in assessing trunk fat mass, the main phenotype in the study. In addition, subjects with cancer were excluded. It is well established that cancer patients usually lose weight [31]. Therefore, application of the cancer exclusion criterion is designed to minimize the influence of the disease on weight-related phenotypes. All subjects who did not complete the 4-year clinical trial were excluded. Among the 155 non-completing subjects, most of the drop-off (n = 128) occurred within the first two years. Because the beneficial effect of calcium on obesity is slow and is not evident in the initial two years, excluding any subjects who did not complete the study is reasonable. After applying the exclusion criteria, we retained 870 qualified subjects. Table 1 lists the number of subjects excluded after applying each exclusion criterion. For the selected 870 subjects, the average calcium compliance rate was 76.0%; the vitamin D compliance rate was 86.9%.

**Table 1:** Subjects selected for the project

Groups		Placebo	Ca-only	Ca + D	Total	*P*
Initial subjects		288	445	446	1179	

Exclusion	Not finished	44	54	58	155	0.46
	Metal prostheses/implants	36	60	52	148	0.71
	Cancer	20	17	13	50	0.03

Total excluded subjects		82	117	110	309	0.52

Applied subjects		206	328	336	870	0.52

**Note: **There were some overlaps between different exclusion criteria.

Not finished: subjects who did not finished the 4-year trial; Metal prostheses/implants: subjects with prosthetics or pacemakers implants; Cancer: subjects suffering from cancer during the 4-year trial.

*P *values were calculated by χ^2 ^test to compare the difference of ineligible subjects among the three groups.

### Phenotypes, biomarker and confounding factors

Phenotypes and biomarkers were measured at baseline (defined as the initial visit before calcium and/or vitamin D intervention), then measured yearly during the 4-year trial (defined as years 1-4). The phenotypes included BMI, trunk fat (TrF), trunk lean (TrL), and percentage of trunk fat (PTrF). Weight and height were measured with subjects' shoes, coats, and other heavy outerwear removed. BMI is calculated by taking a person's weight and dividing by their height squared. TrF and TrL were measured by dual energy x-ray absorptiometry (DXA) using a Hologic 4500C scanner upgraded to a 4500W running software version 8.26 (Hologic Inc., Bedford, MA, USA). The DXA was calibrated each day of analysis, and the margin of error was maintained within 1.5%. Changes in BMI, TrF, TrL, and PTrF (ΔBMI, ΔTrF, ΔTrL, and ΔPTrF) were calculated as the primary outcomes in the present study.

Serum 25-hydroxyvitamin D [25(OH)D] was measured annually. Serum samples for 25(OH)D measurement were collected after a 3-hour fast. Participants were asked not to take vitamin or mineral supplements that morning. Serum 25(OH)D was measured by radioimmunoassay (Nichols/Quest Diagnostics, San Clemente, CA). The coefficient of variation (CV) for intra-assay was 5.1% and inter-assay was 7.9%. All analyses were completed in a single laboratory that participates in the Quality Assurance Program for Vitamin D (DEQAS) [32].

Height, smoking status, and estrogen usage were determined. Age was recorded accurately to one day. Because obesity status may be affected by season, we divided a year into three seasons: a hot season (Jun.-Aug.), a warm/cool season (Mar.-May, Sep.-Nov.), and a cold season (Dec.-Feb.). Smoking status was quantitatively measured as pack-year (amounts previously and currently smoked), and was qualitatively recorded as "never," "former" or "current" smoker. Estrogen usage was recorded as "never," "less than 6 months" and "more than 6 months" of usage.

The total calcium intake included dietary calcium (from food), habitual calcium supplement, and the trial calcium supplementation. Habitual calcium supplementation is defined as "self-selected supplementation that a subject takes during the trial." Trial calcium supplementation was calculated using an assigned dose (1400 mg/d or 1500 mg/d) multiplied by the subject's compliance rate. Calcium compliance rate was measured at 6-month intervals. However, the dietary calcium and habitual calcium supplementation was measured only at baseline visit and at the final visit. To achieve consistent measurements, an average calcium compliance rate was used for calculating the trial calcium intake. The average compliance rate was calculated using all available records during the 4-year trial. Similar to calcium intake, total vitamin D supplementation was measured as a combination of habitual vitamin D supplementation and trial vitamin D supplementation.

### Statistical analysis

Data were analyzed with SPSS for Windows, Release 16.0 (SPSS Inc, Chicago, IL). All tests were two-sided and *P *< 0.05 was considered as significant.

Descriptive statistics and analyses of variance (ANOVA) were performed for confounding factors and phenotypes at baseline. For the follow-up phenotype changes, a Pearson correlation and a stepwise multiple regression were conducted to detect significant confounding factors for each phenotype at each follow-up measure (time point). For the primary analysis, we used an analysis of covariance (ANCOVA) to model the effect of treatment on phenotype changes with significant confounding factors as covariates at each follow-up measure. Independent-sample t-tests applying the Bonferroni correction were performed when the differences between groups were significant. The same analyses were conducted for serum 25(OH)D. In addition to ANCOVA, repeated measures analyses using pooled measures were conducted. In the data analyses, treatment, age, season, estrogen use, and their interactions were included in the model.

For both baseline phenotypes and follow-up phenotype changes, Pearson correlation or Spearman correlation analyses were conducted against various sources of calcium intake and vitamin D supplementation. The same analysis was completed for the baseline serum 25(OH)D.

## Results

### Characteristics of the study subjects

Table 1 summarizes the subjects selected for the study. The initial participants have been described in detail in one of our articles describing their vitamin D status [33]. The following subjects were excluded: 155 subjects who did not finish the study, 148 subjects who had metal prostheses/implants that may affect the accuracy of phenotype measurement, and 50 subjects who were diagnosed with cancer during the study. After applying the exclusion criteria, 870 total subjects were selected for the proposed study. The differences of excluded subjects among the three groups were compared using χ^2 ^test. The *P *values are listed in the Table 1. The excluded subjects among the three groups are not different for any of the tested phenotypes, except for the incidence of cancer.

Table 2 details the basic characteristics of the 870 subjects at baseline. For all baseline characteristics, no significant differences among the three groups emerged. At baseline, the average age of all subjects was 66.0 ± 6.9 y, and average BMI was 28.8 ± 5.3 kg/m^2^. The average 25(OH)D at baseline was 73.2 ± 19.9 nmol/L. The mean total calcium intake of the three groups was 1016 ± 520 mg/d and the mean habitual vitamin D supplementation was 198 ± 189 IU/d.

**Table 2:** Descriptive characteristics (Mean ± SD) for the selected 870 subjects at baseline

Variables	Total *n *= 870	Placebo *n *= 206	Ca-only *n *= 328	Ca + D *n *= 336	*P*
Age (y)	66.0 ± 6.9	65.2 ± 6.5	66.0 ± 6.6	66.5 ± 7.5	0.13
Body mass index (kg/m^2^)	28.8 ± 5.3	28.8 ± 5.3	28.9 ± 5.4	28.7 ± 5.2	0.96
Trunk fat (kg)	14.3 ± 4.7	14.3 ± 4.8	14.5 ± 4.8	14.1 ± 4.6	0.53
Trunk lean (kg)	21.9 ± 3.1	22.1 ± 2.9	22.0 ± 3.2	21.8 ± 3.1	0.37
Percentage of trunk fat (%)	38.6 ± 6.5	38.3 ± 6.6	38.9 ± 6.8	38.5 ± 6.1	0.55
25(OH)D (nmol/L)	73.2 ± 19.9	73.6 ± 20.7	73.0 ± 20.4	73.1 ± 18.8	0.93
Dietary calcium intake (mg/d)	670 ± 389	671 ± 406	681 ± 376	658 ± 392	0.74
Habitual calcium supplement (mg/d)	347 ± 342	334 ± 318	342 ± 354	359 ± 345	0.68
Total calcium intake (mg/d)	1016 ± 520	1004 ± 518	1023 ± 508	1016 ± 535	0.92
Habitual vitamin D supplement (IU/d)	198 ± 189	189 ± 194	206 ± 186	196 ± 188	0.57

Note: *P *values were calculated by analysis of variance (ANOVA) to compare the means of variables among the placebo, Ca-only, and Ca + D groups.

### Test the effects of calcium and calcium plus vitamin D on phenotype changes during the trial

**Figure 1:**
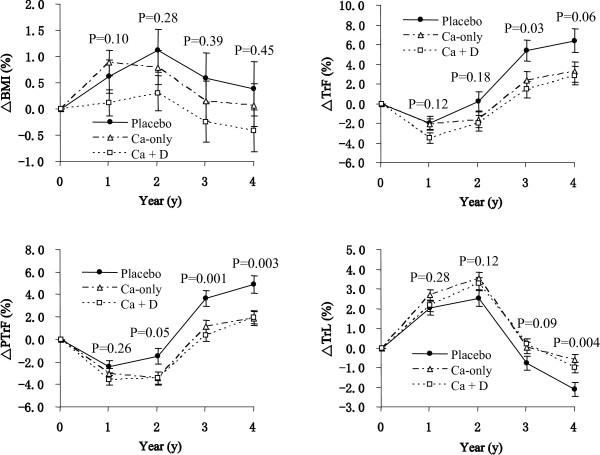
**Percentage changes (Mean ± SE) in body composition by group assignment over the 4 years**. BMI: body mass index, TrF: trunk fat, TrL: trunk lean, PTrF: percentage of trunk fat. Δ: percentage change, equals (Follow-up - Baseline)/Baseline * 100. Ca-only: calcium group; Ca + D: calcium plus vitamin D group; Placebo: placebos group. At baseline (year 0): Placebo (*n *= 206), Ca-only (*n *= 328), Ca + D (*n *= 336); at year 1: Placebo (*n *= 191), Ca-only (*n = *310), Ca + D (*n = *310); at year 2: Placebo (*n *= 187), Ca-only (*n *= 300), Ca + D (*n *= 298); at year 3: Placebo (*n *= 189), Ca-only (*n *= 287), Ca + D (*n *= 296); at year 4: Placebo (*n *= 178), Ca-only (*n *= 274), Ca + D (*n *= 297). The *P *values were calculated by analysis of covariance (ANCOVA) at each follow-up measure (time point). A general linear model (GLM) was used with phenotype changes as dependent variables, treatment as an independent variable, age, season, and estrogen use (stepwise multiple regression *P *< 0.05) as covariates. The significant covariates in the model were as follows: at year 1: season; at year 2: age, season, and estrogen use; at years 3 and 4: age and estrogen use.

**Figure 2:**
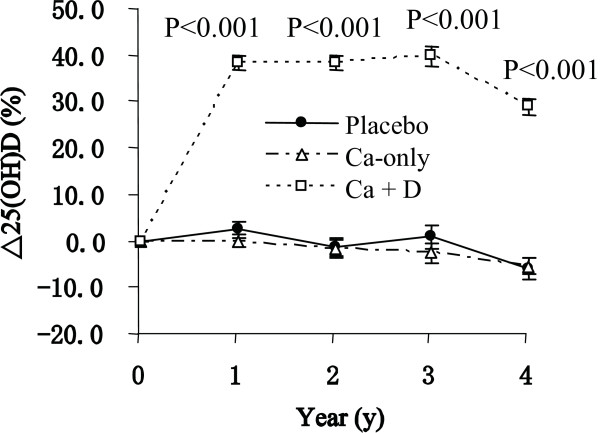
**Percentage changes (Mean ± SE) in serum 25(OH)D by group assignment over the 4 years**. The sample and the data analysis method are the same as Figure 1.

Baseline obesity-related phenotypes are important factors affecting the 4-year obesity status. The effect of calcium intake on obesity-related phenotypes is moderate. In order to reduce the baseline effect and amplify the change of phenotypes, %change is used in Figures 1 and 2. To estimate the absolute change value, this value can be achieved easily by multiplying the percentage change with the basic value in Table 2.

Figure 1 shows the timeline of phenotype changes in the three groups during the 4-year intervention. Analysis of covariance (ANCOVA) was used to test the differences in the three groups. The *P *value is presented in Figure 1 for each year of the study. For ΔBMI, the change trend is different among the three groups, even though significant differences were not observed among the three groups. After 4 years of calcium and vitamin D intervention, the BMI decreased in the Ca + D group, increased in the placebo group, and remained approximately unchanged in the Ca-only group. The weight change is consistent with that of the ΔBMI (data not shown).

Although we did not observe a significant difference among the three groups for ΔBMI, evidence of significant difference was found when we used ΔTrF, ΔPTrF, and ΔTrL. We noted that the changes in TrF and TrL trended in opposite directions. After four years of calcium and vitamin D intervention, subjects from all the three groups gained TrF and lost TrL, although the rates of gain and loss are different between groups. For ΔTrF and ΔPTrF, the Ca-only and the Ca + D groups have lower TrF gain compared to the placebo group (mean 2.4%, 1.4% vs. 5.4%, *P *= 0.015 at year 3), and the effect is more evident for ΔPTrF (mean 1.1%, 0.3% vs. 3.6%, *P *= 0.001 for year 3; and mean 1.8%, 2.0% vs. 4.9%, *P *= 0.003 at year 4). For ΔTrL, Ca-only and Ca + D groups preserve more TrL than the placebo group, and the effect became significant in year four (mean -0.6%, -1.0% vs. -2.1%, *P *= 0.004 at year 4). The results suggest that an increase in calcium intake tends to modify body composition by gaining less TrF and preserving more TrL.

In the study, significant differences were not detected between the Ca-only group and the Ca + D group for any studied phenotypes (Figure 1). This suggests that, in the study cohort, vitamin D supplementation provides no additional beneficial effect on body composition. The correlation analysis provided supportive evidence for this finding. When considering habitual vitamin D supplementation, trial vitamin D supplementation, and total vitamin D supplementation amount, none of them are associated with any changes in the tested phenotypes (Table 3).

**Table 3:** Correlation coefficients between calcium and phenotype changes at year 4 (n = 749)

		ΔBMI	ΔTrF	ΔTrL	ΔPTrF
Calcium	Dietary Ca Intake	0.012	0.007	0.047	-0.002
	Habitual Ca Suppl.	0.053	0.055	0.010	0.058
	Trial Ca Suppl.	-0.037	-0.092*	0.127**	-0.129**
	Total Ca Intake	0.008	-0.034	0.118**	-0.063

Vitamin D	Habitual Vit D Suppl.	0.038	0.060	0.036	0.054
	Trial Vit D Suppl.	-0.066	-0.055	0.008	-0.057
	Total Vit D Suppl.	-0.036	-0.008	0.029	-0.016
	Δ25(OH)D	-0.147**	-0.146**	-0.032	-0.130**

Note: *: *P *< 0.05; **: *P *< 0.01.

Δ: percentage change, equals (Follow-up - Baseline)/Baseline * 100.

BMI: body mass index; TrF: percentage change in trunk fat; TrL: percentage change in trunk lean; PTrF: percentage change in percentage of trunk fat.

Correlation coefficients were calculated by Pearson correlation, using pooled data from the placebo, Ca-only, and Ca + D groups.

After excluding subjects with low adherence (< 80%) of calcium intake, high calcium intake groups still have significantly higher TrL and lower TrF, when compared to the placebo group.

Figure 2 presents the serum 25(OH)D changes in the three groups after calcium and vitamin D intervention. As expected, from baseline to the end of the study, subjects in the Ca + D group had a significant increase in serum 25(OH)D, while the placebo and Ca-only groups remained stable (*P *< 0.001).

We conducted repeated measures analyses that included phenotype measurement time (named as time thereafter), treatment group (named as treatment thereafter), age, season, estrogen usage (named as estrogen thereafter), and their interactions in the model. The results from repeated measures analyses were consistent with those from ANCOVA. For BMI, estrogen and time*age were significant, and the *P *value for treatment was 0.13. For TrF, treatment, age, estrogen, time*treatment, time*age, time*season, and time*estrogen were significant, and the *P *value for treatment was 0.007. For TrL, treatment, season, and time*season were significant, and the *P *value for treatment was 0.01. For PTrF, treatment, age, season, time*treatment, time*age, time*season, and time*estrogen were significant, and the *P *value for treatment was less than 0.001. For 25(OH)D, treatment, season, time*treatment, and time*season were significant, and the *P *value for treatment was less than 0.001.

### Correlations between different types of calcium and vitamin D consumption and baseline phenotypes and year-4 phenotype changes

Results from Figure 1 indicate that calcium supplementation lowers TrF and increases TrL following long-term intervention (three or four years), ultimately modifying body composition. In order to test which type of calcium intake contributes the most beneficial effect, correlations between the amounts of different types of calcium intake and phenotypes were tested at baseline (*n *= 870) (Table 4). At baseline, dietary calcium is not associated with any of the phenotypes (*P *> 0.05). Habitual calcium supplementation was negatively associated with BMI, TrF, TrL, and PTrF (*P *< 0.01). Compared with habitual calcium supplement, total calcium intake had a similar, but weaker, association with all the phenotypes (*P *< 0.01). Table 3 presents the correlation coefficients between the amount of each type of calcium intake and phenotype changes at the end of the study (year 4). Trial calcium supplementation is inversely correlated with ΔTrF and ΔPTrF, and positively correlated with ΔTrL. This result supports the ANCOVA results presented in Figure 1.

**Table 4:** Correlation coefficients between calcium, vitamin D and obesity-related phenotypes at baseline (n = 870)

		BMI	TrF	TrL	PTrF
Calcium	Dietary Ca Intake	-0.054	-0.054	-0.016	-0.065
	Habitual Ca Suppl.	-0.120**	-0.146**	-0.085*	-0.146**
	Total Ca Intake	-0.119**	-0.136**	-0.068*	-0.145**

Vitamin D	Habitual Vit D Suppl.	-0.065	-0.071*	-0.029	-0.093**
	Serum 25(OH)D	-0.260**	-0.294**	-0.179**	-0.281**

Note: *: *P *< 0.05; **: *P *< 0.01.

BMI: body mass index; TrF: trunk fat; TrL: trunk lean; PTrF: percentage of trunk fat.

In the data analysis, the selected subjects in Table 1 were pooled together at baseline. Correlation coefficients were calculated by Pearson correlation.

Serum 25(OH)D was negatively associated with all the phenotypes at baseline (BMI, TrF, TrL, and PTrF, Table 4). Consistently, Δ25(OH)D is inversely correlated with ΔBMI, ΔTrF and ΔPTrF (Table 3). At baseline, habitual vitamin D supplementation levels are associated with TrF and PTrF. The correlation coefficients between habitual vitamin D supplementation and TrF and PTrF are relatively modest when compared to those between serum 25(OH)D and TrF and PTrF.

## Discussion

To the best of our knowledge, this is the first clinical trial in a population-based postmenopausal women cohort, to observe that increasing calcium intake, in the form of non-dairy calcium supplementation, can prevent gain of fat mass and loss of lean mass. The effect of calcium supplementation in this population-based cohort is consistent with the effect of dairy supplementation in fat and lean mass changes in obese subjects with low baseline calcium intake (< 600 mg/d) as reported by Zemel et al. [18-20]. A similar significant finding is reported for body weight in the WHI study in a free-living population of 36282 postmenopausal women [22].

In our study, although we did not find significant differences in the change of BMI among the three groups, we did observe that changes in TrF, TrL, and PTrF are significantly different between the calcium intervention groups and the control group. This may be due to TrF and TrL being more homogenous phenotypes and therefore more sensitive to the effect of calcium.

Figure 1 shows that in all three groups, subjects tended to gain or maintain BMI in the first 2 years. After that, they began to lose BMI in year 3 and 4. This phenomenon is possibly age-related. In a similar study, Caan et al. [22] observed age-related weight change. Postmenopausal women (age ≥ 50) tend to gain and peak their weight in mid to late sixties. Later in life they begin to lose weight. In the present study, the mean ages of the three groups at baseline were 65.2 ± 6.5 (SD), 66.0 ± 6.6 and 66.5 ± 7.5 years old in the placebo, Ca-only, and Ca + D groups, respectively (Table 1). The trajectory of BMI change in our cohort indicates that age ~67 may be the turning point of the effect of age on weight. Considering its potential effects, age was used as a covariate to adjust all studied phenotypes.

The effect of age on BMI (weight) is consistent with its effect on TrL. From Figure 1, it is evident that the subjects in all groups tend to increase TrL in the first year and lose TrL thereafter. The consistent trajectory of BMI and TrL indicates that the change of weight is largely because of the change of TrL. This is compatible with the fact that the main component of total body weight is lean mass.

In contrast to TrL, TrF tends to increase with aging. This phenomenon is consistent with other studies in postmenopausal women [34,35]. Our data indicate that higher calcium intake prevents the accumulation of TrF, and helps to preserve TrL. Lean mass (mostly skeletal muscle) is a key site for energy metabolism. These effects collectively lead to the beneficial effect of reducing the risk of obesity.

In the present study cohort, mean ± SD of baseline calcium intake was 1016 ± 520 mg/d, and the baseline calcium intake for nearly 31% of the subjects was over 1200 mg/d. This is similar to the WHI study (over 39% subjects took over 1200 mg/d calcium). The mean ± SD of baseline 25(OH)D in this study cohort was 73.2 ± 19.9 nmol/L, which is similar to other studies [36,37], but higher than many other US cohorts [38,39].

The average (baseline to year 4) trial calcium supplement was 826 ± 589 mg/d, which is higher than the average dietary calcium (666 ± 323 mg/d) and average habitual calcium supplement (342 ± 314 mg/d). At baseline, habitual calcium supplementation was negatively associated with BMI, TrF, TrL, and PTrF (*P *< 0.01). At the end of the study, trial calcium supplementation is inversely correlated with ΔTrF and ΔPTrF, and positively correlated with ΔTrL. The levels achieved with each type of calcium intake did not affect the correlation analysis.

An interesting finding in this study is that at baseline (Table 4), it is habitual calcium supplementation, not the dietary calcium intake, that contributed to the inverse correlation between BMI, TrF, TrL, PTrF and total calcium intake. This result is similar to that of Gonzalez, et al. [40], who reported an inverse correlation between weight gain and calcium supplementation, but not with dietary calcium intake. On the other hand, others [18,41] have found that dietary calcium has more of an effect on weight than does supplementation. One reason for a lack of effect of dietary calcium is that self-reported dietary calcium intake is difficult to measure.

Clinical trials measuring the effect of vitamin D supplementation on obesity are few. Our study indicates that vitamin D supplementation may have no additional effect on body composition in the presence of high calcium intake. This result is consistent with a previous study conducted by Sneve, et al. [29], which reported that high vitamin D supplementation does not lead to weight loss.

At baseline, consistent with previous reports [26,42,43], we observed that low serum 25(OH)D is negatively associated with BMI, TrF, and TrL. At year 4, Δ25(OH)D is inversely correlated with ΔBMI, ΔTrF and ΔPTrF. These results indicate that serum 25(OH)D may play a role in obesity, although the change in serum 25(OH)D does not linearly reflect the change in trial vitamin D supplementation.

Our study shows that vitamin D supplementation and serum 25(OH)D have different effects on obesity. The different effects may be due to inter-individual differences in the effectiveness of the vitamin D supplementation. The increase of serum 25(OH)D in response to a given dose of vitamin D supplementation is, as reported, widely different from person to person [44]. We found that, after a 12-month vitamin D intervention in our Ca + D group, among the 261 Ca + D subjects who took > 80% vitamin D supplement dosage (i.e. > 880 IU/d) and had serum 25(OH)D measurements at year 1 and baseline, 10 subjects decreased their serum 25(OH)D levels on average by 7% [ie. Δ25(OH)D =-0.07]. Moreover, 251 subjects increased their serum 25(OH)D levels on average by 42% [i.e. Δ25(OH)D = 0.42]. The average vitamin D supplement for the 10 subjects was 1039 IU/d, and for the 251 subjects was 1051 IU/d which was only a little higher than 1039 IU/d.

One strength of this study is that the ethnic backgrounds and living environments of these rural non-Hispanic white women are similar. Relative homogeneity of the population in the parent study is likely to reduce the influence of confounding factors on the measurement of the calcium effect. In addition to the aforementioned strength, we are aware of an extant limitation in this study. Compared with some randomized controlled trials specifically designed for obesity [18-20,22], one limitation of the study is the lack of records of energy intake and physical activity. However, these results are still credible because the population was well-randomized (no significant difference among groups for any confounding factor).

## Conclusion

In summary, study results show beneficial effects of high calcium intake on obesity in a population-based study of postmenopausal women who have a relatively high baseline calcium intake. However, vitamin D supplementation may have no additional effect on body composition.

## Competing interests

The authors declare that they have no competing interests.

## Authors' contributions

JZ, LJZ and QZ participated in the data analysis, interpreted the results, and in the preparation of the manuscript. PW assisted with the data selection and preparation. JML designed the calcium intervention trial, collected the clinical data, assisted with the interpretation of results, and prepared the manuscript. All authors read and approved the final manuscript.
